# Perspectives on Continuing Care, From Home Care to Long‐Term Care, for Older People Living With HIV: A Cross‐Sectional Study

**DOI:** 10.1002/hsr2.70578

**Published:** 2025-03-19

**Authors:** Jacqueline M. McMillan, Jessica Dalere, Farwa Naqvi, Vivian Ewa, Raynell Lang, Raman Brar, Katrina Milaney, Jeffrey A. Bakal, Hartmut B. Krentz, Patrick B. Quail, Caley B. Shukalek, Jenine Leal, Nolan E. Hill, Mark Randall, M. John Gill

**Affiliations:** ^1^ Cumming School of Medicine University of Calgary Calgary Alberta Canada; ^2^ O'Brien Institute for Public Health University of Calgary Calgary Alberta Canada; ^3^ Southern Alberta Clinic Alberta Health Services Calgary Alberta Canada; ^4^ Community Health Sciences University of Calgary Calgary Alberta Canada; ^5^ Provincial Research Data Services Alberta Health Services Edmonton Alberta Canada; ^6^ Data and Research Services Alberta SPOR SUPPORT Unit Calgary Alberta Canada; ^7^ Microbiology, Immunology and Infectious Diseases University of Calgary Calgary Alberta Canada; ^8^ Infection Prevention and Control, Alberta Health Services Calgary Alberta Canada; ^9^ Centre for Sexuality Calgary Alberta Canada; ^10^ Safelink Alberta Calgary Alberta Canada

**Keywords:** aging, continuing care, HIV, home care, long‐term care, older adult, supportive care

## Abstract

**Background and Aims:**

Older (≥ 50 years) persons living with human immunodeficiency virus (PLWH) comprise the majority of individuals receiving HIV care in high‐income countries. PLWH experience the challenges of aging at earlier ages and accelerated rates, compared to people without HIV. Older PLWH who may benefit from more support may hesitate due to fear of stigma, discrimination, or past experiences.

**Methods:**

We assessed the views of older PLWH through an online survey. We sought participants' views, experiences, knowledge and preferences for delivery of continuing care support in Alberta, Canada. Participants were invited during clinic visits at the Southern Alberta Clinic in Calgary, Canada.

**Results:**

A total of 316 participants completed the survey. The mean age was 61 years (standard deviation ± 8) and 83.5% (*n* = 264) were men. Ten percent of participants (*n* = 32) currently receive help with activities of daily living, 70% of which was provided by family/friends. Nine percent expressed concern with receiving help, including financial (54%), loss of independence (31%), and privacy concerns (31%). Nearly 47% expressed concern about healthcare providers' knowledge of HIV, 63% expressed concern about their medical information being kept confidential, and 64% expressed concern about other residents learning of their HIV diagnosis.

**Conclusions:**

Despite a need, many older PLWH are hesitant to accept home care or move into supportive, facility‐based living. This leaves a potential void in the necessary provision of care. This must be addressed to ensure equity, diversity, and inclusion, and to remove barriers in accessing health and social supports. The solutions to this urgent need lie with those with lived experience who can inform healthcare providers and policy‐makers. To start, healthcare providers and policymakers must educate staff about the ongoing stigma and discrimination experienced by older PLWH and demonstrate to patients the value placed on patient privacy and confidentiality.

**Impact Statement:**

We certify that this work is novel clinical research that provides insight into the values and preferences of older people living with HIV regarding continuing care (from home care to supportive living and long‐term care). This work is the foundation for creating meaningful, structural changes to how continuing care is provided to older people living with HIV, an underrepresented and equity‐deserving group. Despite a need, many older PLWH are hesitant to accept home care or move into supportive, facility‐based living. This leaves a potential void in the necessary provision of care. This must be addressed to ensure equity, diversity, and inclusion, and to remove barriers in accessing health and social supports. To start, healthcare providers and policymakers must educate staff about the ongoing stigma and discrimination experienced by older PLWH and demonstrate to patients the value placed on patient privacy and confidentiality.

## Summary

- Nine percent of older PLWH who do not currently receive outside help with activities of daily living expressed concern with receiving help, including financial concern, loss of independence/freedom, privacy concerns, language barriers, stigma, and discrimination.
- Sixty‐four percent of older PLWH expressed worry about their personal medical information being kept private/confidential and about their HIV diagnosis being disclosed to co‐residents, while 47% expressed worry about healthcare providers having sufficient knowledge to care for HIV.
- When considering a move to a facility‐based living environment, older PLWH prioritize: location of the facility, shared/private room, ability to visit family/friends, ability to see their usual HIV doctor/nurse, room size, food/meals, and recreation/activities.

## 1 Introduction

In December of 2023, at the Southern Alberta Clinic (SAC) in Alberta, a province in Canada, 48% of adults receiving treatment for human immunodeficiency virus (HIV) were ≥ 50 years of age. In the United States, at the end of 2022, it was estimated that 42% of persons living with HIV (PLWH) were ≥ 55 years [1]. In 2022, 10% of new HIV diagnoses in the United States were among people ≥ 55 years [1], and in Canada, 6% of new HIV diagnoses were among people ≥ 60 years [2]. In the United States, it is projected that by 2030, 54% of people living with HIV (PLWH) will be ≥ 50 years and 23% will be ≥ 65 years [3]. In HIV, older age is defined as ≥ 50 years [4]. PLWH experiences earlier onset of age‐related conditions such as cardiovascular disease, cancer, cognitive decline, and frailty [5, 6, 7]. The prevalence of age‐related comorbidities in older PLWH is similar to those without HIV who are 10–15 years older [8]. Geriatric syndromes, including frailty, occur at younger ages in PLWH [6, 9], and one‐third of older PLWH have at least mild cognitive deficits [10], which may impair adherence to antiretroviral therapy (ART) [11]. Some studies have found the association between HIV and frailty to be less clear. In a recent study, time since HIV diagnosis was associated with frailty, but duration of ART lowered the odds of frailty [12]. This may be due to survivor bias, an association between untreated HIV and frailty [12], or increased contact with the healthcare system. Frailty is associated with morbidity, hospitalization, long‐term care admission, and death in the general population [13, 14, 15].

Aging PLWH who experience cognitive and/or functional decline that hinders independent living will require additional support provided by continuing care (including home care, supportive/assisted living, and facility‐based care [i.e., long‐term care]). Continuing care is an umbrella term that includes home care and continuing care homes. Home care includes personal and health care delivered within the individual's home, while continuing care homes are congregate living environments where care is provided to individuals with more complex needs that cannot be provided within the individual's home. Continuing care homes range from supportive/assistive living to long‐term care (depending on the amount and complexity of care required) [16]. It is critical that the voices and opinions of older PLWH are incorporated into the provision of these services to create an inclusive environment.

In Canada, proposed policy solutions to address the growing demand for continuing care include placing a greater focus on home care to a lesser extent on long‐term care, in‐line with expressed preferences of many patients [17]. Alberta is the fourth largest province in Canada, with a population of approximately 4.9 million people in 2024 [18]. Alberta's continuing care client profile does not include information on HIV status, and to our knowledge, neither home care utilization nor facility‐based living data for PLWH is currently available in Alberta. Data from 2001 to 2010 in the United States found that the prevalence of PLWH in nursing homes was 1.2% [19]. A more recent picture, in particular, one that reflects the present‐day profile of PLWH in Alberta or, more broadly, in Canada, is not available.

The population of PLWH is aging and will increasingly require home care and facility‐based living to assist in the management of complex physical and medical needs. It is reported that PLWH on ART and with high CD4 T‐cell counts have a near‐normal life expectancy [20]. As Alberta and Canada prepare for the increase in demand for home care and facility‐based living it is critical that the voices of those with lived experience are incorporated into policy and practice change so that care is well‐planned and inclusive. The aim of this work is to synthesize the opinions of older PLWH on home care and facility‐based living through a confidential, online survey of older PLWH conducted between April 1, 2023, and December 31, 2023.

We aimed to: (1) Assess the current knowledge of continuing care and facility‐based living within the province of Alberta; (2) Assess the current and future needs for assistance with activities of daily living; (3) Quantify the current utilization of continuing care and facility‐based living; and (4) Explore perceived barriers and facilitators to accepting the support of continuing care and facility‐based living among older (≥ 50 years) PLWH in Alberta.

## 2 Methods

### 2.1 Study Design

Here, we report on phase 1 of a 3‐phase, cross‐sectional mixed‐methods study. Each phase (including the survey development and pilot testing) has been informed by patient partner(s) with lived experience to ensure inclusive language and that priority topics are covered. The survey was available in English as well as the 10 most commonly spoken languages among older PLWH at SAC (Supporting Information S1: Appendix 1). The survey was pilot‐tested to ensure that the directions, language, and content were clear and inclusive.

*Phase 1*. Between April 1 and December 31, 2023, older PLWH were invited to participate in the 24‐question online survey during a routine clinic visit (Supporting Information S2: Appendix 2). An iPad was provided to complete the survey during the visit. Trained research personnel were available to assist participants if needed. For patients receiving virtual care, an invitation to participate, with a QR code, was provided during regular pharmacy visits when medications were dispensed.

### 2.2 Setting

The SAC provides centralized HIV care to all PLWH in southern Alberta, Canada. As of April 16, 2024, 1277 patients (47.6%) were ≥ 50 years of age. More than one‐third of older PLWH at SAC report living alone, and based on our routine frailty assessments, approximately 14% of PLWH ≥ 50 years at SAC are frail [21].

### 2.3 Participants

Using this longitudinal, well‐characterized, and geographically defined cohort, we invited patients who were ≥ 50 years of age to complete an online survey. The final sample was a convenience sample of PLWH attending routine clinic visits who consented to participate. Recruitment was through posters and verbal invitations to participate. We collaborated with community partners, including the Center for Sexuality and Safelink Alberta, during the conception, planning, recruitment, and data synthesis for this work.

Inclusion criteria: ≥ 50 years of age and receiving HIV care at SAC.

Exclusion criteria: < 50 years of age. Recruitment was only completed at SAC, and SAC exclusively provides HIV care; therefore, people without HIV were not eligible for recruitment.

Consent was obtained before survey completion. We encouraged recruitment that was diverse and inclusive of different age groups, sex, gender, sexual orientation, race/ethnicity, and socioeconomic status.

### 2.4 Predictor Variables

Participants self‐reported their demographic information, including age, gender, sexual orientation, geographic location, current living situation (i.e., living alone, living with others, living in a congregate environment, unstably housed or homeless), and whether they have previously, or were currently receiving home care or residing in facility‐based living.

### 2.5 Outcomes

In Alberta, continuing care includes home care, supportive/assisted living, and facility‐based living (i.e., long‐term care). Participants' knowledge of the care continuum and options for continuing care in Alberta were assessed using an ordinal scale (Supporting Information S2: Appendix 2). Participants' acceptance of support services was asked (e.g., willing/unwilling). Participants were asked what types of assistance in activities of daily living (if any) they were currently receiving or which they felt would be of benefit to them (e.g., basic activities of living, including assistance to bath, dress, walk, transfer, toilet, eat; and/or instrumental activities of daily living [IADLs], including assistance with medication management, meal preparation, cleaning, laundry, shopping, transportation). Perceived barriers and potential enablers to engaging in continuing care, were explored. Potential responses included lists of options, ordinal scale responses, as well as open text responses.

### 2.6 Statistical Analysis

Descriptive statistics were utilized to calculate means, standard deviations, medians, and interquartile ranges for continuous data, and frequencies and proportions for categorical data. Survey response free text data was analyzed qualitatively and, where applicable, were categorized within existing groups, or added as unique responses. All statistical tested were conducted on SAS 9.4.

### 2.7 Ethical Approval

This study was approved by the University of Calgary Conjoint Health Research Ethics Board (CHREB) REB22‐1222.

Study results are presented in accordance with STROBE (STrengthening the Reporting of OBservational studies in Epidemiology) [22].

## 3 Results

Three‐hundred and sixteen participants completed the online survey. During the study period (April 1–December 31, 2023), there were 576 unique in‐person clinic appointments, and 462 patients were invited to complete the survey for a response rate of 68% (316/462). Participants were not obligated to respond to all questions to advance in the survey, so for some questions, there were fewer than 316 responses.

### 3.1 Demographic Characteristics

As displayed in Table 1, the mean age of survey respondents was 61 years (standard deviation ± 8.2, range 50–83 years), and the median age was 60 years (interquartile range 54, 66). The majority were men (*n* = 264, 84%), aged 50–59 years (*n* = 147, 46%) (Table 1). Most participants identified as gay (*n* = 165, 52%), heterosexual/straight (*n* = 108, 34%), or bisexual (*n* = 21, 6.7%) (Table 1).

**Table 1:** Demographic data of 316 survey participants recruited from the Southern Alberta HIV Clinic (SAC) between April 1 and December 31, 2023.

Characteristic	*N* = 316 (%)
Age (years)	
50–59	147 (47%)
60–69	118 (37%)
70+	50 (16%)
Gender	
Man	264 (84%)
Woman	49 (15%)
Two‐Spirit (Indigenous)	3 (0.95%)
Transgender man	0 (0.00%)
Transgender woman	0 (0.00%)
Nonbinary	0 (0.00%)
Gender‐fluid	0 (0.00%)
Another gender identity	0 (0.00%)
Sexual identity (more than one may apply)	
Gay	165 (52%)
Heterosexual/straight	108 (34%)
Bisexual	21 (6.6%)
Prefer not to answer	17 (5.4%)
Asexual	11 (3.5%)
Another (please specify)	3 (0.95%)
Pansexual	2 (0.63%)
Queer	2 (0.63%)
Two‐Spirit (Indigenous)	2 (0.63%)
Lesbian	0 (0.00%)
Living location	
Calgary	279 (88%)
Small community outside of Calgary or Edmonton (e.g., Airdrie, Cochrane, High River)	19 (6.0%)
A city other than Calgary or Edmonton (e.g., Red Deer, Medicine Hat, Lethbridge)	8 (2.5%)
Rural or remote community (e.g., Bassano, Long View, Two Hills)	10 (3.2%)
Edmonton	0 (0.00%)
Living with	
I live with one other person	124 (39%)
I live alone	119 (38%)
I live with two or more other people	66 (21%)
I live with several other people in a shared setting (e.g., seniors' apartment, lodge, supportive living, or long‐term care)	7 (2.2%)
If living with others, with whom do you live (more than one may apply)	
Spouse or partner(s)	90 (46%)
Friend(s)	36 (18%)
Spouse or partner(s) AND children	26 (13%)
Other family (e.g., siblings, parents, other relatives)	26 (13%)
Children only	12 (6.1%)
Strangers (e.g., I currently live in a seniors' apartment building, lodge, supportive living, or long‐term care)	11 (5.6%)
Dwelling type	
House	127 (40%)
Apartment	88 (28%)
Condo	45 (14%)
Townhome	36 (11%)
Basement suite	10 (3.2%)
Emergency sheltered (no stable or permanent home)	3 (0.95%)
Supportive living	3 (0.95%)
Lodge	2 (0.63%)
Unsheltered/homeless	1 (0.32%)
Long‐term care	0 (0.00%)
Own or rent residence	
Rent	155 (49%)
Own	145 (46%)
Neither	16 (5.1%)

The majority of respondents lived in Calgary at the time of survey completion (*n* = 279, 88%). Most respondents lived with one other person (*n* = 124, 39%), or alone (*n* = 119, 38%), while only 7 lived with several other people in a shared setting (e.g., seniors' apartment, lodge, supportive living, or long‐term care) (2.2%). For those who reported living with others, most (*n* = 90; 46%) lived with a spouse or partner. Most participants lived in a house (*n* = 127, 40%) and rented their home (*n* = 155, 49%) (Table 1).

### 3.2 Participants' Current Support and Self‐Reported Need for Support

**Figure 1:**
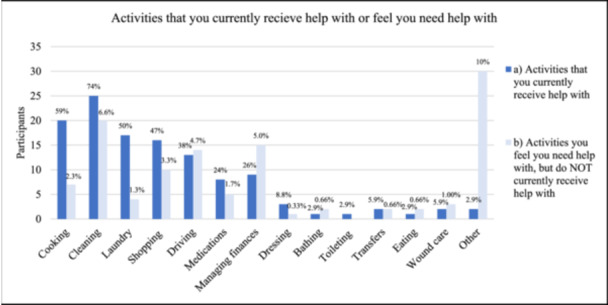
Activities that you currently receive help with or feel you need help with. (a) Of those currently receiving help, activities they are currently receiving help with (*n* = 33). (b) Of all respondents, activities they feel they need help with but are not currently receiving (*n* = 115). “Other” responses: exercise, foot/toenail care, heavy luggage, housing, job search, and physiotherapy.

Supporting Information S3: Appendix 3 and Figure 1 present data on participants' current support and self‐reported need for support. Ten percent (*n* = 32) of participants received help for activities of daily living (e.g., cooking, cleaning, laundry, dressing, bathing, taking medications, or wound care). For the majority of those receiving help, the help was provided by the family (*n* = 17, 52%) (Supporting Information S3: Appendix 3). Less commonly, help was provided by friends, hired help (paid for privately), Home Care, or nurses in their facility (Supporting Information S3: Appendix 3). The activities that participants received help with included cleaning (*n* = 25, 74%), cooking (*n* = 20, 59%), laundry (*n* = 17, 500%), shopping (*n* = 16, 47%), driving (*n* = 13, 38%), managing finances (*n* = 9, 27%), and medications (*n* = 8, 24%) (i.e., IADLs); as well as, dressing (*n* = 3, 8.8%), transfers (*n* = 2, 5.9%), wound care (*n* = 2, 5.9%), bathing (*n* = 1, 2.9%), toileting (*n* = 1, 2.9%), and eating (*n* = 1, 2.9%) (i.e., basic activities of daily living [BADLs]) (Figure 1).

When asked, “Are there activities that you feel you need help with but which you do not currently receive help,” 62 (21%) said yes, and provided the following responses: cleaning (*n* = 20, 6.6%), driving (*n* = 14, 4.7%), managing finances (*n* = 15, 5.0%), shopping (*n* = 10, 3.3%), cooking (*n* = 7, 2.3%), medications (*n* = 5, 1.7%), and laundry (*n* = 4, 1.3%) (i.e., IADLs), followed by wound care (*n* = 3, 1.0%), transfers (*n* = 2, 0.7%), eating (*n* = 2, 0.7%), bathing (*n* = 2, 0.7%), and dressing (*n* = 1, 0.3%) (i.e., BADLs) (Figure 1).

As shown in Supporting Information S4: Appendix 4, at the time of survey completion, 10 participants (3.2%) were receiving Home Care, 15 (4.7%) had received home care in the past, 9 (2.8%) lived in a seniors' apartment, 4 (1.3%) lived in supportive living, and 1 (0.3%) lived in a lodge. None of the participants were living in a long‐term care facility at the time of survey completion (Supporting Information S4: Appendix 4).

As shown in Supporting Information S5: Appendix 5, when asked, “Please select the ONE option that best describes your plans and preferences as you age,” 159 participants (51%) stated “I would like to stay in my own home, but if I need extra help I will accept it”; 84 participants (27%) stated “I would like to stay in my own home, without outside help”; 33 participants (11%) stated “When the time is right, I will move into a lodge, supportive‐living or long‐term care”; and 36 participants (12%) were unsure (Supporting Information S5: Appendix 5).

### 3.3 About the Resources Available in Alberta

As shown in Supporting Information S6: Appendix 6, most participants indicated that they were “not at all familiar” with the supportive care available to them as an Albertan (*n* = 144, 46%), 26 (8.3%) were extremely familiar, 33 (11%) were moderately familiar, 54 (17%) were somewhat familiar, 46 (15%) were slightly familiar (Supporting Information S6: Appendix 6), and 11 (3.5%) were unsure (Supporting Information S6: Appendix 6). The majority (*n* = 281, 90%) indicated that a healthcare provider had not previously suggested that they would benefit from additional support, while 16 (5.1%) indicated that it had been suggested.

As shown in Supporting Information S7: Appendix 7, of participants not currently receiving outside help, 34 (11%) felt that they would benefit from it, 187 (60%) did not feel they would benefit, 77 (25%) felt they might benefit, and 8 (2.6%) preferred not to say. Of participants not currently receiving outside help, 29 (9.3%) had concerns or hesitation about receiving help, 212 (68%) did not, 58 (19%) were unsure, and 7 (2.3%) preferred not to say (Supporting Information S7: Appendix 7).

**Figure 2:**
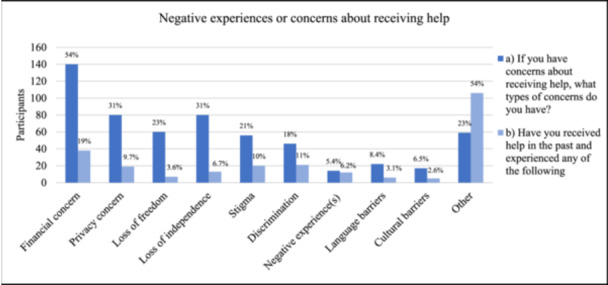
Negative experiences or concerns about receiving help. (a) If you have concerns about receiving help, what types of concerns do you have (*n* = 261), (b) have you received help in the past and experienced any of the listed concerns (*n* = 195)?

Figure 2 shows participants' responses when asked to select all concerns that apply to them regarding receiving help, the following concerns were identified: financial (*n* = 140, 54%), loss of independence (*n* = 80, 31%), privacy (*n* = 80, 31%), loss of freedom (*n* = 60, 23%), fear of stigma (*n* = 56, 22%), fear of discrimination (*n* = 46, 18%), language barriers (*n* = 22, 8.4%) and negative experience(s) in the past (*n* = 14, 5.4%) (Figure 2).

Participants who had received help in the past reported experiencing the following: financial concern (*n* = 38, 20%), stigma (*n* = 20, 10%), discrimination (*n* = 21, 11%), privacy concern (*n* = 19, 9.7%), loss of independence (*n* = 13, 6.7%), negative experience(s) (*n* = 12, 6.2%), loss of freedom (*n* = 7, 3.6%), language barriers (*n* = 6, 3.1%), and cultural barriers (*n* = 5, 2.6%) (Figure 2).

**Figure 3:**
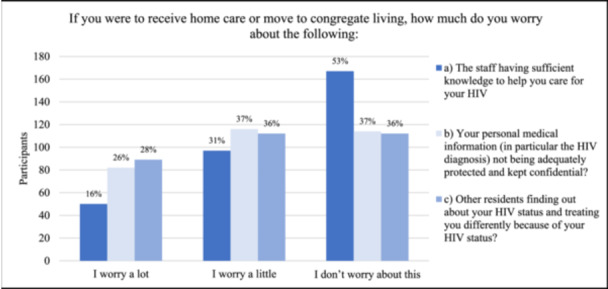
If you were to receive home care or move to congregate living, how much do you worry about: (a) the staff having sufficient knowledge to help you care for your HIV (*n* = 314); (b) your personal medical information not being adequately protected and kept confidential (*n* = 312); (c) other residents finding out about your HIV status and treating you differently because of your HIV status (*n* = 313)?

Figure 3 shows participants' responses when asked, “If you were to receive home care or move to congregate living (lodge, supportive living facility, or long‐term care) how much do you worry about the staff having sufficient knowledge to help you care for your HIV?” Fifty (16%) worried a lot, 97 (31%) worried a little, and 167 (53%) did not worry (Figure 3). When asked how much they would worry about their personal medical information (in particular the HIV diagnosis) not being adequately protected and kept confidential, 82 worried a lot (26%), 116 worried a little (37%), and 114 (37%) did not worry at all (Figure 3). When asked how much they would worry about other residents finding out about their HIV status and treating them differently because of their HIV status, 89 worried a lot (28%), 112 worried a little (36%), and 112 did not worry at all (36%) (Figure 3).

**Figure 4:**
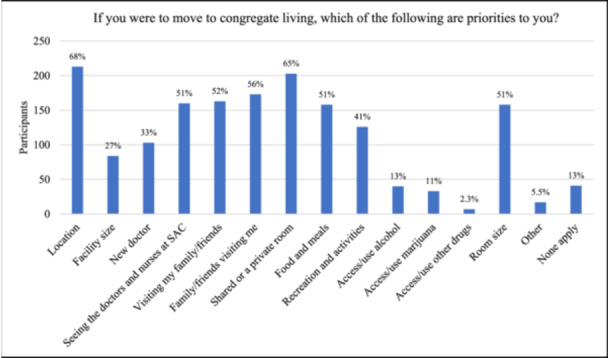
If you were to move to congregate living, which of the following are priorities to you? (*n* = 311).

Figure 4 shows participants' responses when asked about their priorities if they were to move to congregate living (lodge, supportive living facility, or long‐term care); participants most commonly indicated: the location of the facility (*n* = 213, 69%), shared/private room (*n* = 203, 65%) whether their family/friends could visit them (*n* = 173, 56%), whether they could visit their family/friends (*n* = 163, 52%), whether they could still see the doctors and nurses at SAC (*n* = 160, 52%), room size (*n* = 158, 51%), the food/meals (*n* = 158, 51%), and recreation/activities (*n* = 126, 41%) (Figure 4).

## 4 Discussion

The aim of this cross‐sectional study was to synthesize the opinions of older PLWH on home care and facility‐based living through an online survey. Participants in this survey ranged in age from 50 to 83 years, and 84% were male, with over 60% self‐reporting as members of the LGBTQ community. Gender identity and gender roles significantly influence where and with whom one lives, as well as the type and frequency of support one may receive. The majority of participants in this study currently dwell in urban centers, however, nearly 6% are residents of rural and remote communities. We believe that the representativeness of this sample provides a powerful voice to historically underserved equity‐deserving members of the community.

As of April 16, 2024, 47.6% of patients at SAC were ≥ 50 years, and one‐third of older PLWH reported living alone, which, along with female sex, is associated with having unmet care needs [23]. At SAC, 14% of older PLWH are frail [21], which, in the general population, is associated with long‐term care admission [13, 15]. For older PLWH accepting home care and facility‐based living may cause apprehension. Further to this, there is a void of knowledge of the current profile of either home care recipients or facility‐based living residents living with HIV. To create more inclusive continuing care options, the voices of those with lived experience, including their needs, and opinions on barriers and facilitators, must be incorporated into future planning.

Ten percent of participants in the present study reported receiving outside help, of whom 70% receive help from family and/or friends and 18% receive help from Home Care or facility‐based staff. In contrast, in 2021, among the general Canadian population, 6% of households reported use of formal care in the preceding year (5.3% in Alberta) [24]. In the general population, informal assistance in the prior year was reported by 14% of Canadians aged ≥ 65 years in 2020 [25]. Those who lived alone (19%) were more likely to receive informal care than those living with others (12%), and the assistance was provided by someone within the same household in 42% and from someone living outside of the household in 58% of respondents [25]. The proportion of Canadians ≥ 85 receiving informal assistance was over three times higher than those aged 65–84 years (38% vs. 11%) [25]. It is necessary for healthcare providers and policymakers to be aware of the substantial amount of informal and unpaid support that family and friends provide to older PLWH and to provide the necessary resources to assist them.

The majority of participants in the present study were familiar with the supportive care options available to them as Albertans (51%), while 46% were not at all familiar. Our search of the literature does not have comparative numbers for the general population. Efforts should be made to educate and inform older PLWH on the supportive care options in their region to ensure that health and personal care services are delivered efficiently to those in need of them.

Nine percent of participants in the present study who do not currently receive outside help expressed concern about receiving this help. They expressed concerns unrelated to their HIV diagnosis (e.g., financial, loss of independence/freedom, privacy, and language barriers) as well as concerns related to their HIV diagnosis (e.g., stigma and discrimination). A smaller proportion of participants had previously experienced each of the above concerns. That 10% of participants have experienced stigma and/or discrimination during a period of receiving outside help is unjust and unacceptable. Older PLWH deserve access to safe and welcoming continuing care options. It is imperative for healthcare providers and policymakers to be informed of the impact of stigma and discrimination on older PLWH, and policies need to be implemented to welcome and affirm older PLWH within all healthcare environments.

Housing status is associated with treatment access and health outcomes for PLWH in the United States [26], yet many older PLWH are hesitant to engage in the support provided by continuing care due to stigma, historical trauma, and fear of an unwelcoming or unsafe environment [27]. A recent review explored the intersectionality of HIV‐ and aging‐related stigma among older PLWH [28], and the 2021 United Nations Political Declaration on HIV and AIDS encouraged a particular focus on the delivery of care to older PLWH that is free of stigma and discrimination [29].

Financial concerns were the most commonly expressed in the present study. Given that Home Care and facility‐based living are covered in Alberta by the Alberta Health Insurance Plan (AHIP) and other provincial plans across Canada, this may reflect an opportunity for further education, especially as 46% of participants were not at all familiar with the supportive care options available to them. This may be related to the relatively young age of the sample population.

Sixty‐four percent of participants in the present study expressed either a lot or a little worry about their personal medical information being kept confidential and about their HIV diagnosis being disclosed to co‐residents and being treated differently as a result. Nearly half expressed worry about healthcare providers having sufficient knowledge to care for HIV. Combined, this represents a large proportion of significant and valid concerns related to their privacy, confidentiality, and trust in the system, which, as a result, may impose barriers to accessing needed care in the near future. Healthcare providers and the health system broadly, should demonstrate clearly and explicitly the value and priority placed on the protection of patients' privacy and confidentiality.

## 5 Limitations

This study has several limitations. The first is related to the sample population and the use of a convenience sample which may limit the generalizability of the findings. The majority of participants lived in Calgary, a large urban center, which may not be representative of the views of older PWLH in rural and remote communities. Participants who do not attend regular, in‐person medical appointments may have been missed during recruitment. Despite this, we still achieved a 68% survey response rate. Language barriers or limited experience with technology may have limited recruitment. However, we minimized this through assistance from trained research personnel, as well as translation of the survey into the 10 most commonly spoken languages among older adults at SAC. This study relied on self‐reported data, which is vulnerable to recall and social desirability bias. Lastly, the surveys are limited in their ability to garner context and additional meaning from participants' responses. We have recently completed 1:1 interviews with a diverse sample of older PLWH to deepen and broaden our understanding of their values, perspectives, and suggestions for the delivery of continuing care to older PLWH. Over 100 of the 316 survey participants expressed an interest in participating in these interviews, reflecting a powerful desire for engagement from the community.

## 6 Conclusion

The results of this work provide opportunities for policy and practice change for PLWH who receive home care or who reside in facility‐based care in Alberta. To start, healthcare providers and policymakers must educate staff of the ongoing stigma and discrimination experienced by older PLWH and demonstrate to patients the value placed on patient privacy and confidentiality.

This work is the foundation for future work aimed at creating meaningful, structural changes to how home care and facility‐based living are provided to older PLWH. This will provide a framework to advocate for policy and practice change at the local, provincial, and national levels. We have begun these discussions and knowledge mobilization strategies aimed at sharing the results with policy‐ and decision‐makers locally. Due to their unique lived experiences, it is imperative that aging PLWH are represented as policies are considered and implemented to address their future needs.

## Author Contributions

**Jacqueline M. McMillan:** conceptualization, data curation, funding acquisition, investigation, methodology, project administration, supervision, writing – original draft, writing – review and editing. **Jessica Dalere:** conceptualization, data curation, project administration, writing – review and editing. **Farwa Naqvi:** conceptualization, data curation, project administration, writing – review and editing. **Vivian Ewa:** conceptualization, investigation, methodology, writing – review and editing. **Raynell Lang:** conceptualization, investigation, methodology, writing – review and editing. **Raman Brar:** data curation, formal analysis, methodology, writing – review and editing. **Katrina Milaney:** conceptualization, investigation, methodology, writing – review and editing. **Jeffrey A. Bakal:** conceptualization, investigation, methodology, writing – review and editing. **Hartmut B. Krentz:** conceptualization, investigation, methodology, writing – review and editing. **Patrick B. Quail:** conceptualization, investigation, methodology, writing – review and editing. **Caley B. Shukalek:** conceptualization, investigation, methodology, writing – review and editing. **Jenine Leal:** conceptualization, investigation, methodology, writing – review and editing. **Nolan E. Hill:** conceptualization, investigation, methodology, writing – review and editing. **Mark Randall:** conceptualization, investigation, methodology, writing – review and editing. **M. John Gill:** conceptualization, investigation, methodology, writing – review and editing. All authors have read and approved the final version of the manuscript. Jacqueline M. McMillan and Raman Brar had full access to all of the data in this study and take complete responsibility for the integrity of the data and the accuracy of the data analysis.

## Conflicts of Interest

The authors declare no conflicts of interest.

## Transparency Statement

The lead author, Jacqueline M. McMillan, affirms that this manuscript is an honest, accurate, and transparent account of the study being reported; that no important aspects of the study have been omitted; and that any discrepancies from the study as planned (and, if relevant, registered) have been explained.

## Supporting information

Supporting information.

Supporting information.

Supporting information.

Supporting information.

Supporting information.

Supporting information.

Supporting information.

## Data Availability

The participants of this study did not give written consent for their data to be shared publicly, so due to the sensitive nature of the research supporting data is not available.
