# Supplementary material for: Perspectives on Continuing Care, From Home Care to Long‐Term Care, for Older People Living With HIV: A Cross‐Sectional Study
Source: Health Sci Rep. 2025 Mar 19;8(3):e70578. doi: 10.1002/hsr2.70578 (PMC11922802; doi:10.1002/hsr2.70578)
Supplement: Supplementary file 1 — Supporting information. [file HSR2-8-e70578-s004.docx]

**Appendix 1.** Top 10 most commonly spoken languages among the intended study population of older PWH at SAC (in addition to English language)

1. Tigrinya
2. Amharic
3. Punjabi
4. Cantonese
5. French
6. Spanish
7. Arabic
8. Russian
9. Burmese
10. Shona
