# Supplementary material for: Perspectives on Continuing Care, From Home Care to Long‐Term Care, for Older People Living With HIV: A Cross‐Sectional Study
Source: Health Sci Rep. 2025 Mar 19;8(3):e70578. doi: 10.1002/hsr2.70578 (PMC11922802; doi:10.1002/hsr2.70578)
Supplement: Supplementary file 2 — Supporting information. [file HSR2-8-e70578-s002.docx]

**Appendix 2. Perspectives on Continuing Care for older persons with HIV Survey**

Participants will have read and agreed to the consent form which provides background information prior to starting the survey.

SECTION 1. ABOUT YOU AND WHERE YOU LIVE

1. What is your current age?
2. What is your current gender identity?

- Man
- Woman
- Transgender man
- Transgender woman
- Two-Spirit (Indigenous)
- Non-binary
- Gender-fluid
- Another gender identity (please specify) ________________________
- Prefer not to answer

1. Do you identify as (choose all that apply):
   - Asexual
   - Bisexual
   - Gay
   - Heterosexual/straight
   - Lesbian
   - Pansexual
   - Queer
   - Two-Spirit (Indigenous)
   - Another (please specify) _________________________
   - Prefer not to answer
2. Where do you currently live?
   - Calgary
   - Edmonton
   - Small community outside of Calgary or Edmonton (e.g. Airdrie, Cochrane, High River)
   - A city other than Calgary or Edmonton (e.g. Red Deer, Medicine Hat, Lethbridge)
   - Rural or remote community (e.g. Bassano, Long View, Two Hills)
3. How many other people do you currently live with?
   - I live alone
   - I live with one other person
   - I live with two or more other people
   - I live with several other people in a shared setting (e.g. seniors apartment, lodge, supportive living or long-term care)
4. If you live with others, with whom do you live?
   - Spouse or partner(s)
   - Spouse or partner(s) AND children
   - Children only
   - Other family (e.g. siblings, parents, other relatives)
   - Friend(s)
   - Strangers (e.g. I currently live in a seniors apartment building, lodge, supportive living or long-term care)
5. What type of dwelling do you live in?
   - Apartment
   - Condo
   - Townhome
   - House
   - Basement suite
   - Emergency sheltered (no stable or permanent home)
   - Unsheltered/homeless
   - Lodge
   - Supportive living
   - Long-term care
6. Do you currently own or rent your home?
   - Own
   - Rent
   - Neither

SECTION 2. ABOUT YOUR CURRENT SUPPORT AND NEED FOR SUPPORT

1. Do you currently receive help (family/friends or professional help) for daily activities (e.g. for cooking, cleaning, doing the laundry, dressing, bathing, taking medications, wound care)?
   - Yes
   - No
   - Unsure
2. If you currently receive help, who provides the help? Select all that apply:
   - Family
   - Friends
   - Home Care
   - Hired help (paid for privately)
   - Nurses in the facility where I live
   - Unsure
3. If you currently receive help, please check the activities that you currently receive help with (please check all that apply):
   - Cooking
   - Cleaning
   - Laundry
   - Shopping
   - Driving
   - Medications
   - Managing finances
   - Dressing
   - Bathing
   - Toileting
   - Getting into/out of a chair or bed
   - Eating
   - Wound care
   - Other ________________________________
4. Are there activities that you feel you need help with, but which you do NOT currently receive help with (please check all that apply):
   - Cooking
   - Cleaning
   - Laundry
   - Shopping
   - Driving
   - Medications
   - Managing finances
   - Dressing
   - Bathing
   - Toileting
   - Getting into/out of a chair or bed
   - Eating
   - Wound care
   - Other ­­­­­­­­­­­­­­­­­­­­­_____________________________________
5. Please select from the following, if they apply to you:
   - I currently receive home care
   - I have received home care in the past
   - I currently live in a seniors’ apartment
   - I currently live in a lodge
   - I currently live in supportive living
   - I currently live in a long-term care
   - None of the above applies to me
6. Please select the ONE option that best describes your plans and preferences as you age:
   - I would like to stay in my own home, without outside help
   - I would like to stay in my own home, but if I need extra help I will accept it
   - When the time is right, I will move into a lodge, supportive living or long-term care
   - Unsure

SECTION 3. ABOUT THE RESOURCES AVAILABLE IN ALBERTA

1. How familiar are you with the supportive care available to you as an Albertan (e.g. home care, supportive living, long-term care):
   - Extremely familiar
   - Moderately familiar
   - Somewhat familiar
   - Slightly familiar
   - Not at all familiar
   - Unsure
2. Has a health care provider ever suggested to you that you would benefit from additional support (e.g. home care, supportive living, long-term care)?
   - Yes
   - No
   - Unsure
   - Prefer not to say
3. If you do NOT currently receive help, do you feel that you would benefit from outside help?
   - Yes
   - No
   - Maybe
   - Prefer not to say
4. If you do NOT currently receive help, do you have concerns or hesitation about receiving this type of support?
   - Yes
   - No
   - Unsure
   - Prefer not to say
5. If you have concerns about receiving help, what types of concerns do you have? (Please select all that apply)
   - Financial concern
   - Privacy concern
   - Loss of freedom
   - Loss of independence
   - Fear of stigma
   - Fear of discrimination
   - Negative experience(s) in the past
   - Language barriers
   - Cultural barriers
   - Other (space will be limited to one line for this response) ________________
6. Have you received help in the past and experienced any of the following (please select all that apply):
   - Financial concern
   - Privacy concern
   - Loss of freedom
   - Loss of independence
   - Stigma
   - Discrimination
   - Negative experience(s)
   - Language barriers
   - Cultural barriers
   - Other (space will be limited to one line for this response) ________________
7. If you were to receive home care or move to congregate living **(*lodge, supportive living facility or long-term care)***, how much do you worry about the staff having sufficient knowledge to help you care for your HIV?
   - I worry a lot
   - I worry a little
   - I don’t worry about this
8. If you were to receive home care or move to congregate living **(*lodge, supportive living facility or long-term care)***, how much do you worry about your personal medical information (in particular the HIV diagnosis) not being adequately protected and kept confidential?
   - I worry a lot
   - I worry a little
   - I don’t worry at all
9. If you were to receive home care or move to congregate living **(*lodge, supportive living facility or long-term care)***, how much do you worry about other residents finding out about your HIV status and treating you differently because of your HIV status?
   - I worry a lot
   - I worry a little
   - I don’t worry at all
10. If you were to move to congregate living **(*lodge, supportive living facility or long-term care)***, which of the following are priorities to you (please select all that apply):
    - Where the facility is located
    - How big the facility is
    - Who my new doctor is
    - Whether I can still see the doctors and nurses at Southern Alberta Clinic (SAC)
    - Whether I can visit my family/friends
    - Whether my family/friends can visit me
    - Whether I have a shared room or a private room
    - The food and meals
    - The recreation and activities
    - Whether I am able to access and use alcohol
    - Whether I am able to access and use marijuana
    - Whether I am able to access and use other drugs
    - Room size
    - Other ____________________________
