# Supplementary material for: Perspectives on Continuing Care, From Home Care to Long‐Term Care, for Older People Living With HIV: A Cross‐Sectional Study
Source: Health Sci Rep. 2025 Mar 19;8(3):e70578. doi: 10.1002/hsr2.70578 (PMC11922802; doi:10.1002/hsr2.70578)
Supplement: Supplementary file 4 — Supporting information. [file HSR2-8-e70578-s005.docx]

**Appendix 4.** Which of the following apply to you? (Current home care/housing status (n=317)
