# Supplementary material for: Perspectives on Continuing Care, From Home Care to Long‐Term Care, for Older People Living With HIV: A Cross‐Sectional Study
Source: Health Sci Rep. 2025 Mar 19;8(3):e70578. doi: 10.1002/hsr2.70578 (PMC11922802; doi:10.1002/hsr2.70578)
Supplement: Supplementary file 7 — Supporting information. [file HSR2-8-e70578-s001.docx]

**Appendix 7.** If you do NOT currently receive help: a) Do you feel you would benefit from outside help (n=306); b) Do you have concerns or hesitation about receiving this type of support (n=306)?
